# Microencapsulated Linseed Oil Supplementation Modifies Lipid Profile and Improves Luteal Function in Dairy Sheep

**DOI:** 10.1002/fsn3.70097

**Published:** 2025-04-25

**Authors:** Ignacio Contreras‐Solís, Cristian Porcu, Francesca Daniela Sotgiu, Valeria Pasciu, Neda Todorova, Laura Mara, Fabrizio Chessa, Margherita Addis, Myriam Fiori, Giovanni Molle, Maria Dattena, Antonio Gonzalez‐Bulnes, José Alfonso Abecia, Fiammetta Berlinguer

**Affiliations:** ^1^ Veterinary Medicine Department Sassari University Sassari Italy; ^2^ AGRIS Sardegna Sassari Italy; ^3^ Veterinary Faculty Cardenal Herrera University Valencia Spain; ^4^ Veterinary Faculty Zaragoza University Zaragoza Spain

**Keywords:** ewes, fertility, linseed oil, lipid‐metabolism status, ovarian function

## Abstract

Polyunsaturated fatty acids omega 3 (PUFA‐ω3) have been shown to modulate reproductive events such as ovarian follicular and luteal development, steroid and prostaglandin synthesis, and oocyte/embryo quality in different species. These effects could be exploited to support pregnancy and avoid early embryo losses that could occur in dairy sheep breeding. This study aimed to evaluate the effectiveness of dietary supplementation of microencapsulated/by‐passed linseed oil (LO) on ovarian function, embryo implantation rates, and lipid profiles of Sarda ewes during their early pregnancy. Our results demonstrated that the intake of microencapsulated LO at a level of 4.0% of fresh matter increased the plasmatic concentrations of PUFASω3 (*p* < 0.01) and progesterone (*p* < 0.05), as well as cholesterol (*p* < 0.01), triglyceride (*p* < 0.001), high‐density lipoprotein (*p* < 0.001), and non‐esterified fatty acids (*p* < 0.05). The percentage of ewes in estrus, ovulation rate per mated ewe, number of embryos per ewe, and pregnancy rates were similar between treated and control groups. In conclusion, dietary supplementation of by‐pass LO during the preimplantation period increased PUFAS‐ω3 distribution at systemic and local levels. Also, this supplementation modified the ewe's lipid profile and improved luteal function with a possible positive effect on embryo‐maternal crosstalk and embryo implantation rate during and after the maternal recognition of pregnancy.

## Graphical abstract

Linseed oil supplementation on lipid‐metabolism status. Linseed oil supplementation on ovarian function.

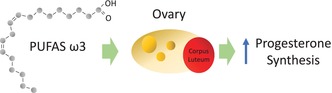

## 1 Introduction

Embryo losses have been an interesting scope of study in livestock species because of their impact on reproductive efficiency and productivity in ruminant production systems (Diskin and Morris 2008). In this sense, the development of methods to enhance reproductive output of ewes may positively impact farm profitability while reducing environmental impacts. Reducing reproductive losses is a first step toward this aim. In ewes, most pregnancy losses arise during the early stages of pregnancy—the first 14 days (O'Connell et al. 2016) and are estimated around 12%–55% (Diskin and Morris 2008). In particular, approximately one‐third of the losses occurs by Day 4, with the rest occurs between Days 4 and 14 (O'Connell et al. 2016). Most ewes that fail to become pregnant do not show a delay in the expression of estrus, indicating that the embryo was lost prior to maternal recognition of pregnancy (Quinlivan et al. 1966). These events are mainly attributed to genetic problems (e.g., breed male/female effect or chromosomal defect; VanRaden and Miller 2006), maternal physiological status (e.g., milking yield level or age; McMillan and McDonald 1985; Diskin and Morris 2008) and environmental factors (e.g., thermal stress, health, nutritional status; Diskin and Morris 2008). In the latter case, nutritional factors such as adequate maternal nutritional status and/or strategic supplementation or use of nutraceutical products have been an important focus of study to avoid or reduce embryo losses in ruminants (Viñoles et al. 2012; Kruse et al. 2017). Ewes and cows fed with an adequate supply of dry matter showed reduced embryo losses and increased pregnancy rates compared with underfed females (Diskin et al. 2016; Meikle et al. 2018). The use of alternative energy sources, such as fatty acids, has also proved to have a positive impact on pregnancy rates in ovine, bovine, and swine females (Zeng et al. 2023). Fatty acid supplementation can enhance luteal function, in terms of progesterone secretion, thus supporting early embryo development in sheep (Mirzaei‐Alamouti et al. 2018) and cattle (Giller et al. 2018; Leroy et al. 2014).

Polyunsaturated fatty acids omega 3 (PUFA‐ω3) and omega 6 (PUFA‐ω6) play an important role in several physiological events, such as growth, brain development, vision, and reproduction in different mammalian species. In domestic ruminants, they have been shown to modulate reproductive events, such as ovarian follicular and luteal development, steroid and prostaglandin synthesis, and oocyte/embryo quality (Mattos et al. 2000; Wathes et al. 2007; Gulliver et al. 2012; Elis et al. 2016).

Among different fatty acids from botanical origin, alpha linolenic acid (ALA) has been recognized as a remarkable source of PUFA‐ω3. Its importance is also attributed to its effects on reproduction and milk quality in dairy cows (Wathes et al. 2007; Petit and Benchaar 2007; Moallem 2018). In fresh pastures, the ALA levels vary between 0.6% and 3.2% (Kalač and Samková 2010). However, factors such as plant maturitys stage, seasonal changes, and biohydrogenation at the ruminal level determine its availability (Jenkins and Harvatine 2014). Linseed oil (LO) is considered the main source of ALA used in large and small ruminant species (Moallem 2018; El‐Tarabany et al. 2020). Currently, the use of microencapsulated LO has been a useful tool to assure the bioavailability/absorption of LO at the intestinal level due to the fact that this protected form avoids its degradation/biohydrogenation at the ruminal level (Jenkins and Bridges 2007).

Earlier research studies have demonstrated the ability of LO to enhance ovarian follicular growth, ovulatory, and fertility rates in dairy cattle (Zachut et al. 2010; Pereira et al. 2022). Moreover, LO showed to have a luteoprotective effect (Gulliver et al. 2012), which could be partly attributed to the positive relationship among fatty acid intake, serum cholesterol, lipoproteins, and progesterone (P_4_). In beef cows, the intake of by‐pass mixed (saturated and unsaturated) long‐chain fatty acids (Hawkins et al. 1995) has indeed been linked with increased circulating levels of cholesterol, high‐density lipoproteins, and P_4_. This association is positively linked with dietary fat levels such as has been found in prepubertal goats (Bomfim et al. 2016). Nevertheless, there are other reports that do not confirm these findings in ruminants, which suggest further investigations about this issue (Moallem 2018; Gulliver et al. 2012). Moreover, the effect of bypass LO on ovarian function and lipid metabolism during early pregnancy needs to be elucidated, given its potential impact on embryo implantation.

Starting from these premises, the current study was designed to determine whether dietary use of microencapsulated/by‐pass LO during early pregnancy can increase follicular and luteal function (in terms of estradiol and progesterone production) and implantation rates in ewes. The bioavailability of by‐pass LO was investigated by assessing lipid metabolism before, during, and after supplementation.

## 2 Material and Methods

The study was carried out at the Agricultural Research Agency of Sardinia (AGRIS)‐Experimental Farm, Bonassai (40°40′ 24″ N. and 8°21′ 59.4″ E.), Sardinia, Italy, from October to December 2020. The experimental procedures were approved by Italian Health Ministry‐Ethic Committee (Authorization n°757/2020‐PR/Protocol‐E8652.2).

### 2.1 Experimental Design

Forty dry Sarda ewes (0.95 ± 0.21 parturitions per ewe) were allocated to two homogeneous experimental groups: LO‐treatment group (*N* = 20), which was nourished using a diet enriched with microencapsulated LO (60 g of ALA microencapsulated at 18% rate in a pelleted concentrate, SILA, Verona, Italy), and CT‐control group (*N* = 20), which was fed with a LO‐free diet. The experimental period lasted 46 days, including 10 days of adaptation (PRE period) from day −26 to −17 preceding mating (Day 0) to adapt the ewes to the new environment and dietary regimens. The full diet feeding period lasted 36 days and consisted of three periods: (1) 2 weeks before the mating period (BMP; from day −16 to −2; weeks −2 to −1); (2) 2 days of synchronized mating period (days −1 and 0); and (3) 3 weeks after the mating period (AMP; from days +1 to +21; weeks +1 to +3).

### 2.2 Body Weight and Body Condition Score

At the onset of the experimental period (PRE; Day −26), both CT and LO ewes were balanced in terms of age (CT = 2.46 ± 0.54 years old; LO = 2.38 ± 0. 49 years old; *p* = 0.882), BW (CT = 39.99 ± 1.15 kg; LO = 38.37 ± 1.03 kg; *p* = 0.299) and BCS (CT = 2.58 ± 0.08; LO = 2.56 ± 0.08; *p* = 0.802). Body weight (BW) was obtained using an electronic scale. Body Condition Score (BCS) was recorded according to Russel et al. (1969). Both BW and BCS were measured and registered at the onset of the PRE period. Then, these were recorded weekly during the full diet feeding period (weeks −2 to +3). These measures were carried out before feeding (from 08:00 to 09:00 h).

### 2.3 Feedstuff Composition and Intake Measurement

CT and LO diets were formulated to obtain similar protein (isoproteic) and energy (isoenergetic) content (Table 1). The CT diet consisted of pelleted concentrate (300 g/ewe day) mixed with barley (250 g/ewe day), while the LO diet was formulated using pelletized concentrate (250 g/ewe day), mixed with barley (120 g/ewe day), and microencapsulated LO (60 g/ewe day). Both CT and LO diets were offered individually—one time a day—between 8:00 and 10:00. Then, the remaining food was weighed and registered (except for mating period; MP) to determine the individual intake.

**TABLE 1:** Composition of experimental diets in ewes non supplemented (CT) and supplemented with by‐pass linseed oil (LO).

Total nutrients (%/Kg DM)	CT	LO
DM	87.89	88.32
CP	9.12	8.29
NDF	55.28	57.05
ADF	30.54	32.04
ADL	2.86	3.11
EE	2.01	5.08
Starch	12.17	8.00
Ash	9.42	10.08
Total NE (Mcal/ewe/day)	1.66	1.65

Abbreviations: ADF, acid detergent fiber; ADL, acid detergent lignin; CP, crude protein; DM, dry matter; EE, ether extract; NDF, neutral detergent fiber; NE, net energy.

Fiber component consisted of 1125 g of hay ( *Lolium multiflorum* )/ewe/day, which was added to each experimental diet. To evaluate the variability of hay intake within the treatment group, animals from both experimental groups were assigned to three pens (total six sub‐groups; three/experimental group). Hay was offered to each subgroup, and residuals were measured 24 h later to estimate daily intake during the full diet feeding period (except for MP).

### 2.4 Estrous Synchronization

Estrous and ovulations were synchronized in the CT and LO groups to obtain a synchronized luteal development. Thus, intravaginal sponges containing 20 mg fluorogestone acetate (Chronogest, MSD, France) were inserted for 14 days (coinciding with weeks −2 and −1 from experimental period). In addition, 350 UI/ewe of equine chorionic gonadotropin (Folligon, MSD, the Netherlands) was administered when the pessaries were removed. All ewes (CT and LO groups) were exposed to fertile rams 48 h after the removal of the pessaries Rams were marked with a colored crayon in order to identify sheep in estrus during the mating period.

### 2.5 Blood Sampling

Blood samples were taken weekly (07:00 h) during the experimental period (from Day −24, −14, −7, +1, +8, +14) for each ewe. EDTA (VACUTEST KIMA, Italy) vacuum tubes were used to collect blood samples to measure cholesterol, triglycerides, low‐density lipoprotein (LDL), high‐density lipoprotein (HDL), and non‐esterified fatty acids (NEFA). Also, blood samples were taken using lithium heparinized (VACUTEST KIMA, Italy) vacuum tubes to measure fatty acid methyl esters.

Blood samples were taken at Days −2 (pessary removal), −1, and 0 (mating) using lithium heparinized vacuum tubes to measure estradiol (E_2_). In addition, blood samples were collected on Days 5, 8, 11, 14, and 16 (after mating) using EDTA vacuum collection tubes to measure progesterone (P_4_). Then, these samples were centrifuged at 3000 rpm for 15 min, and plasma was stored at −20°C until assayed.

### 2.6 Fatty Acids Methyl‐Esters ( *FAMES* ) Analyses

Samples taken on Days −24 and 0 were analyzed to determine fatty acid levels in plasma. Plasma total lipids were extracted according to the method described by Jiang et al. (1996). Aliquots (1 mL) of plasma were transferred into a glass centrifuge tube, and 1.8 mL of isopropanol was added. After vigorous shaking, 1.3 mL of n‐hexane was added, and the mixture was homogenized using a vortex (Velp Scientifica Srl, Italy) for 3 min. The mixture was then centrifuged at (1094 *g*) for 10 min at 4°C, and the upper layer was transferred to a second glass test tube. The lower layer was extracted twice with 1.3 mL of n‐hexane, and the supernatants were pooled with the previous n‐hexane layer. The n‐hexane layer was evaporated with a rotary evaporator at 30°C. The extracted fat was stored at −20°C until further analysis. The extracted lipids were subjected to acid transesterification (Chin et al. 1992; Stanton et al. 1997). The gas chromatographic separation of fatty acid methyl esters (FAMEs) was achieved using the Agilent 8890 GC System (Santa Clara, CA) on a capillary column Supelco SP 2560 (100 m length, 0.25 mm inner diameter, 0.2 μm film thickness). Helium was used as the carrier gas at a flow rate of 1 mL/min. The injection was performed in split mode, and the split ratio was 1:20. One microliter of FAME sample was injected under the following gas chromatography conditions: the oven temperature was programmed at 45°C and held for 4 min, increased to 175°C at a rate of 13°C/min, held for 27 min, increased to 215°C at a rate of 4°C/min, and held for 35 min. The injector and detector (flame ionization detector) temperatures were set at 290°C. Individual FAMEs were identified by comparison to a standard mixture of 37 components (Matreya Inc., Pleasant Gap, PA) and by published isomeric profiles (Kramer et al. 2004). The quantitative measurement of each FAME was performed through a calibration curve using the following internal standards C13:0 (C10:0–C17:0) and C19:0 (C18:0–C18:3). The concentration of each internal standard added to the fat sample was 170 mg/g of lipid.

### 2.7 Metabolic Status

Blood plasma samples were analyzed to determine circulant levels of cholesterol, triglycerides, LDL, HDL, and NEFA. They were quantified using commercial kits and a clinical chemistry analyzer previously described (Contreras‐Solís et al. 2023). Sensitivities from different metabolites were 3 mg/dL (for cholesterol, and triglycerides), 2 mg/dL (for LDL and HDL), and 0.01 mmol/L (for NEFA). Intra‐and interassay coefficient variations (CV) for cholesterol, triglycerides, LDL, HDL, and NEFA were 1.3%, 0.99%, 1.0%, 1.55%, and 1.07%, and 1.24%, 1.24%, 1.60%, 1.75%, and 1.15%, respectively.

### 2.8 Hormonal Assay

Blood samples from Days −2 to 0 were used to determine E_2_ concentrations in plasma. They were measured using an estradiol‐17β commercial ELISA kit (Demeditec Diagnostics GmbH, Kiel‐Wellsee, Germany) used for ovine species (Bruno‐Galarraga et al. 2021). Sensitivity and intra‐assay CV were 1.40 pg/mL and 5.5%, respectively.

Also, samples from days 5, 8, 11, 14, and 16 were used to determine P_4_ circulating concentrations in plasma. Progesterone levels were measured using a P_4_ ELISA kit (DiaMetra S.R.L., Perugia, Italy) validated by Pasciu et al. (2022) for ovine species. The sensitivity, intra‐and interassay CV were 0.05 ng/mL, 4%, and 9.3%, respectively.

### 2.9 Ultrasonographic Scanning

Presence and number of corpora lutea (CL) at days 5 and 8 post‐mating (Day 0) were determined using a transrectal ultrasonographic scanner (US). Also, US was used to determine the presence and number of embryos at Day 24 post‐mating. Pregnancy was diagnosed by the presence of large uterine horn—filled with anechoic liquid—and embryo vesicles.

All procedures were carried out using the US (Model ProSound 2 V, HITACHI‐ALOKA Medical Ltd., Japan) fitted with a linear probe (7.5 MHz transducer; UST‐660‐7.5, Aloka Co., Japan).

### 2.10 Statistical Analyses

The assumption of normal distribution and homogeneity of variance was analyzed using Wilk–Shapiro and Levine's test, respectively. Non‐parametric tests were performed when assumptions were not accomplished. Age and BCS at the onset of the experimental period were analyzed using the non‐parametric Mann–Whitney *U* test, and BW was analyzed using one‐way ANOVA.

Intake, BW, BCS, E_2_, P_4_, cholesterol, triglycerides, LDL, HDL, and NEFA concentrations during the experimental period (week/day) were analyzed using repeated measures ANOVA‐General linear model (GLM) using the following factors: treatment, week/day, and their interaction. Bonferroni post hoc was used to determine differences among different factors. Considering the energetic content for both diets, the intake was calculated and expressed in terms of percentage of DM intake and Mcal of net energy. The percentage of FAMES was analyzed using univariate analyses.

Bivariate (correlation) analyses were carried out to determine the association between: (a) fatty acids intake (in terms of total EE intake) and cholesterol, triglycerides, and NEFA levels in plasma; (b) LDL and cholesterol; (c) LDL and triglycerides; (d) HDL and cholesterol; and (e) cholesterols levels and P_4_ progesterone data after MP, was determined by correlation analyses.

One ewe was withdrawn from reproductive data analyses due to the presence of an ovarian cyst. The number of CL and embryos was analyzed using the non‐parametric Mann–Whitney *U* test. Estrus and pregnancy rates were analyzed using the Fisher non‐parametric test.

Statistical data analyses were performed using RStudio. A Bonferroni post hoc test was performed to determine differences among fixed factors. Statistical differences and tendencies were established at 5% (*p* < 0.05) and 10% (*p* < 0.10), respectively.

## 3 Results

In our study, no differences between groups in BCS and BW appeared during the experimental period (from −2 to +3 Weeks). However, BW was affected (*p* < 0.001) by the time/week factor (Table 2). Individual daily DM intakes of concentrate and hay, expressed as a % of the respective daily offer, were homogeneous between experimental groups (*p* = 0.885 and *p* = 0.739 for hay and concentrate, respectively; Table 2). Hence, net energy intake, calculated from total daily intake (g DM/ewe/day), did not differ between groups during the experimental period (Table 2).

**TABLE 2:** Body weight (BW), body condition score (BCS), percentage of dry matter intake (% DM/ewe/day) and net energy intake (NE; Mcal/ewe/day) from concentrate and hay during experimental period between ewes fed with a control diet (CT; *n* = 20) and ewes supplemented with by‐pass linseed oil (LO; *n* = 20).

	Groups		*p*		
	CT (Mean ± S.E.M.)	LO (Mean ± S.E.M.)	Group	Week	Group × Week
BW (Kg)	40.94 ± 0.48	39.36 ± 0.42	0.320	0.000	0.054
BCS^a^	2.62 ± 0.03	2.57 ± 0.03	0.565	0.213	0.643
Concentrate DM intake as % of daily offer	93.12 ± 1.16	93.62 ± 1.29	0.885	0.002	0.374
Hay DM intake as % of daily offer^b^	82.39 ± 1.39	83.02 ± 1.23	0.739	0.001	0.950
NE concentrate intake (Mcal/ewe/day)	0.78 ± 0.01	0.77 ± 0.01	0.614	0.002	0.344
NE hay intake (Mcal/ewe/day)^b^	0.64 ± 0.01	0.64 ± 0.01	0.916	0.139	0.942

Abbreviation: S.E.M., standard error of the mean.

^a^ Score 1–5.

^b^ Estimated individual intake.

The plasma level of saturated (SFA), unsaturated (UFA), mono‐unsaturated (MUFA), and PUFAS‐ω3 and ω6 was homogeneous between experimental groups on the starting day of the trial (Table 3). LO supplementation led to significant changes, as on Day 0 circulating levels of PUFAS‐ω3 were higher in LO compared to CT ewes (*p* < 0.001) while MUFA levels were lower in the LO group (*p* < 0.05; Table 3). SFA, UFA, and PUFAS‐ω6 were similar between groups (Table 3).

**TABLE 3:** Percentage of Fatty acids methyl ester (FAMES; Mean ± S.E.M.) in plasma at Days −26 (PRE period) and 0 (Mating period) in ewes fed with a control diet without by‐pass linseed oil (CT; *n* = 20) and supplemented with by‐pass linseed oil (LO; *n* = 20).

% FAMES	Groups			
	CT	LO	CT	LO
	PRE period (Day −26)		Mating period (Day 0)	
SFA	42.37 ± 1.40	43.77 ± 0.85	42.30 ± 1.42	45.30 ± 1.46
UFA	57.64 ± 1.40	56.23 ± 0.85	57.70 ± 1.42	54.70 ± 1.46
MUFA	24.81 ± 1.72	22.27 ± 1.54	25.69 ± 1.67^a^	18.41 ± 1.65^b^
PUFAS‐ω3	5.30 ± 0.20	5.89 ± 0.32	5.66 ± 0.61^c^	11.35 ± 0.42^d^
PUFAS‐ω6	27.53 ± 0.82	28.08 ± 0.96	26.35 ± 0.99	25.00 ± 0.67

*Note:* a, b superscripts indicate differences at *p* < 0.05. c, d superscripts indicate differences at *p* < 0.001.

Abbreviations: Mating period, mating during synchronized estrus; MUFA, monounsaturated fatty acids; PRE, onset of experimental period; PUFAS‐ω3, polyunsaturated fatty acids omega 3; PUFAS‐ω6, polyunsaturated fatty acids omega 6; SFA, saturated fatty acids; UFA, unsaturated fatty acids.

**FIGURE 1:**
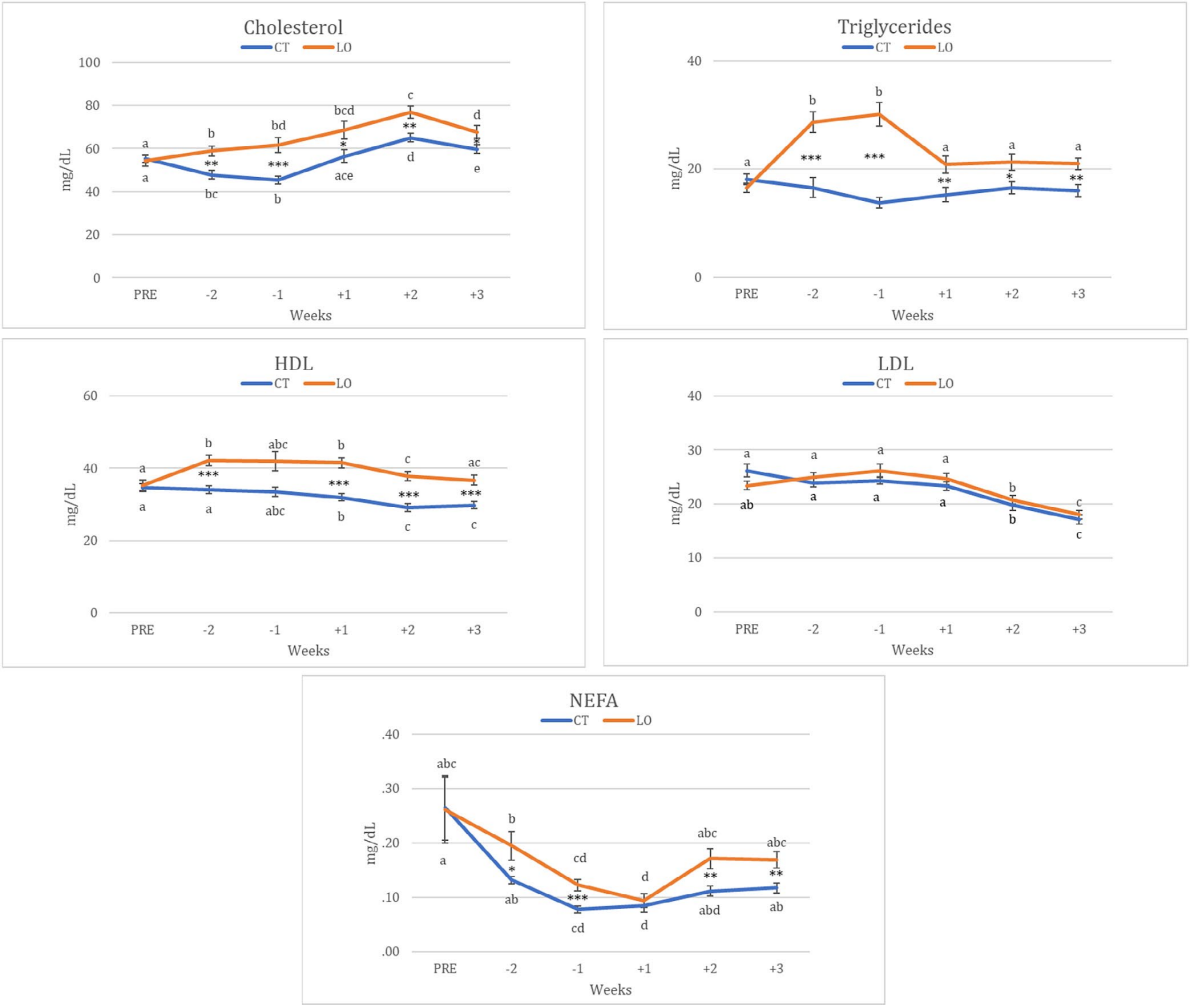
Circulating levels of cholesterol, triglycerides, low density lipoprotein (LDL), high density lipoprotein (HDL), and non‐esterified fatty acids (NEFAs) in ewes fed with a control diet (CT; *N* = 20) and ewes supplemented with by‐pass linseed oil (LO; *N* = 20). *, **, *** indicate differences between groups at *p* < 0.05, *p* < 0.01 and *p* < 0.001, respectively. a, b, c, d, and e indicate significant differences within the same group. PRE: Onset of experimental period; Week−2 and −1: Experimental feeding period prior to mating; Weeks from+1 to+3: Experimental feeding period after mating.

Blood metabolic parameters did not differ between groups in the pre‐experimental period. Thereafter, cholesterol and triglycerides plasma levels reached higher values in LO ewes compared to CT ones (*p* < 0.01 and *p* < 0.001, respectively; Figure 1), as well as HDL (on week −2 and from week +1 to +3; *p* < 0.001) and NEFAs (weeks −2, −1, +2 and + 3: *p* < 0.05). Circulating concentrations of low‐density lipoprotein were not statistically different between groups.

Total EE intake was positively correlated with total cholesterol (*r* = 0.612; *p* = 0.01) and triglycerides (*r* = 0.648; *p* = 0.001; Table S1). Moreover, there was a moderate and positive association between HDL and cholesterol (*r* = 0.556; *p* = 0.001). However, there was a very weak association between LDL and triglycerides (*r* = 0.198; *p* = 0.01; Table S1).

**FIGURE 2:**
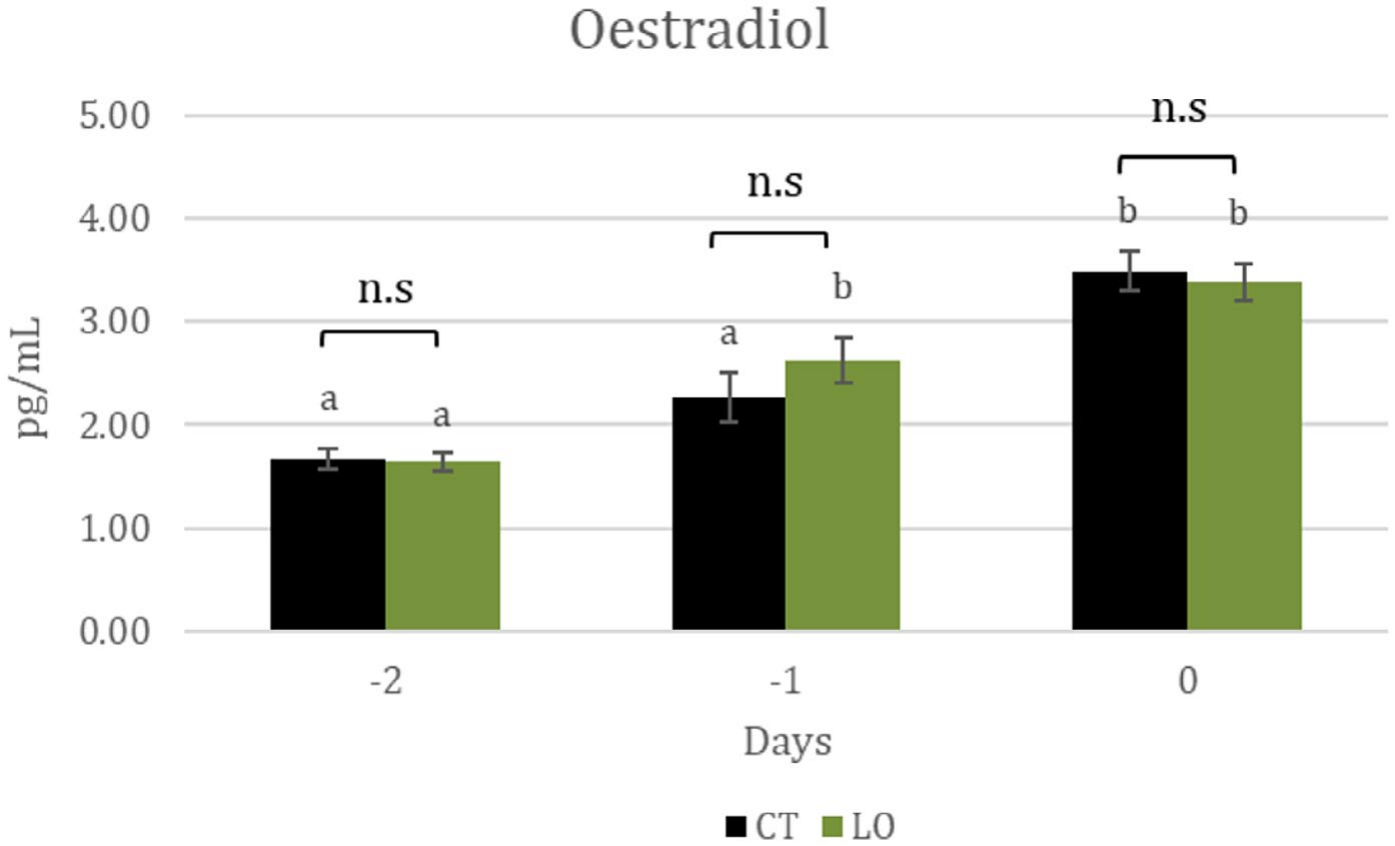
Plasmatic estradiol concentrations (pg/mL) from Day −2 (timing pessary withdraw) to Day 0 (mating) in ewes non supplemented (CT; *N* = 20) and supplemented with by‐pass linseed oil (LO; *N* = 19). Superscripts (a,b) indicates differences among days in the same groups (*p* <  0.05).

Estradiol level during the induced follicular phase increased from Days −2 (pessary withdrawal) to 0 (mating) in both groups. However, estradiol level was not different between the groups (Figure 2).

**FIGURE 3:**
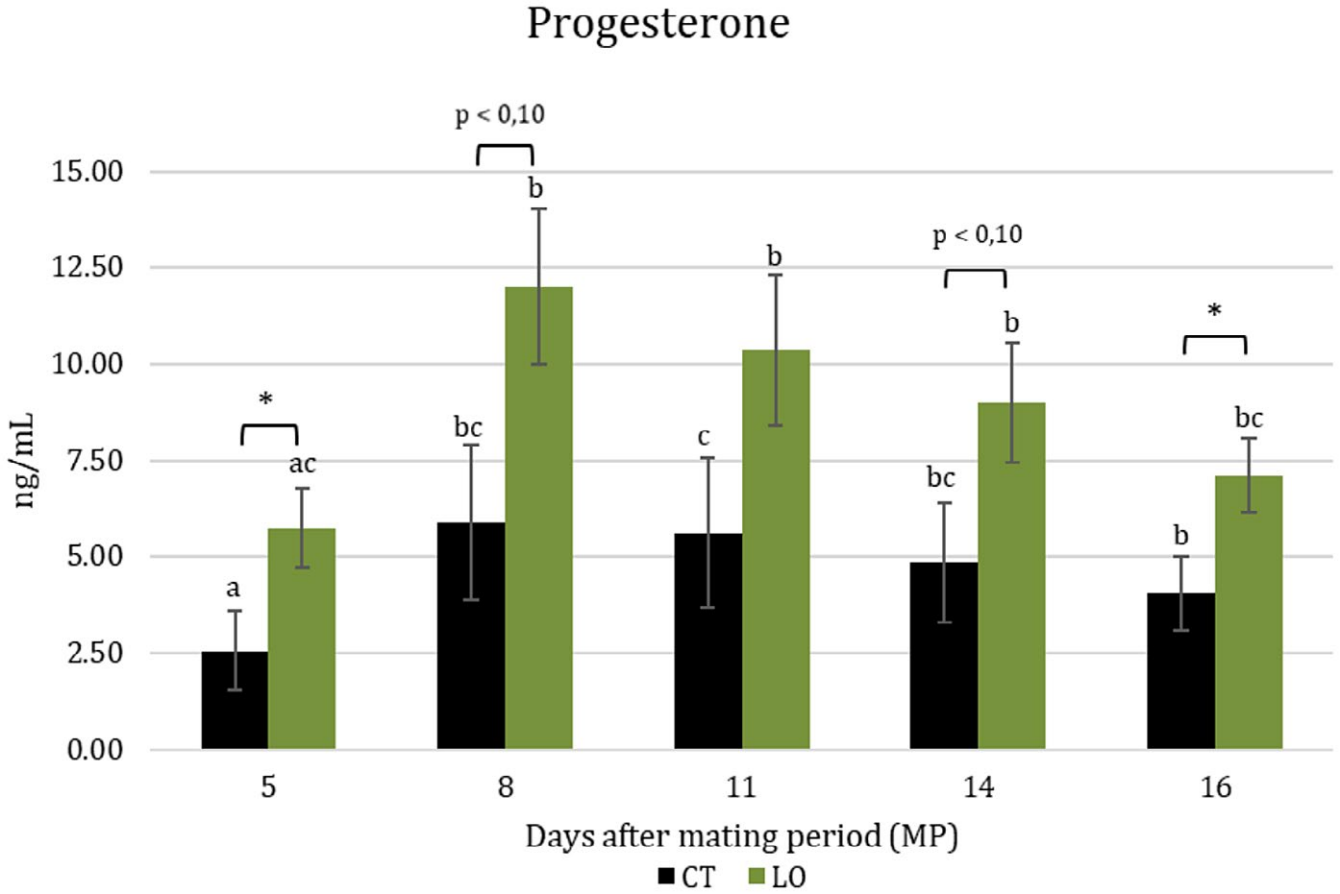
Circulating progesterone concentrations (pg/mL) at Days 5, 8, 11, 14 and 16 (after 67 mating) in ewes non supplemented (CT; *N* = 20) and supplemented with by‐pass linseed oil (LO; 68 *N* = 19). Superscripts indicates statistical differences among days into the same groups (*p* < 69 0.05). *p* < 0.10 and asterisk indicate tendency and statistical differences (*p* < 0.05), respectively, 70 between CT and LO groups.

Overall P_4_ concentrations from Days 5 to 16 were higher in LO ewes (CT = 5.45 ± 0.42 vs. LO = 7.59 ± 0.72 ng/mL; *p* < 0.05). Moreover, there was a trend (*p* < 0.10) toward an increase in P_4_ concentration in LO groups on Days 8 and 14, coincident with the timing of maternal recognition of pregnancy (Figure 3). Despite differences between cholesterol and P_4_ levels between CT and LO groups on days 8 (week +2) and 14 (week +3) after mating, the correlation between these variables was not significant (*r* = 0.093, *p* = 0.495).

The number of ewes in estrus and pregnancy rates were similar between experimental groups (Table 4). All ewes from the LO group showed estrus under controlled mating during the induced follicular phase. Only 3 (15%) ewes from the CT group did not show estrus. Also, the number of CL per ewe after estrus and per pregnant ewe, and the number of total embryos and the number of embryos per ewe were not statistically different for the CT and LO groups.

**TABLE 4:** Summary of reproductive traits between ewes fed with a control diet (CT; *n* = 20) and supplemented with by‐pass linseed oil (LO; *n* = 19).

	CT	LO
Ewes in estrus	17/20 (85%)	19/19 (100%)
N° CL/ewe in estrus	1.88 ± 0.15	1.94 ± 0.21
Pregnant ewes/ewes mated	15/20 (75.0%)	17/19 (89.5%)
Pregnant ewes/ewes in estrus	15/17 (88.2%)	17/19 (89.5%)
N° CL/pregnant ewe	1.93 ± 0.15	2.00 ± 0.21
Total embryos (ewes in estrus)	23 (*n* = 17)	29 (*n* = 19)
N° embryos/pregnant ewe	1.53 ± 0.13/ewe (*n* = 15)	1.71 ± 0.17/ewe (*n* = 17)

*Note:* N° CL/ewe in estrus: number of corpora lutea per ewe in estrus. Pregnant ewes/ewes in estrus: number/percentage of pregnant ewes per ewe mated. Pregnant ewes/ewes in estrus: number/percentage of pregnant ewes per ewe in estrus. N° CL/Pregnant ewe: number of corpora lutea per pregnant ewe. Total embryos (ewes in estrus): total number of embryos from ewes in estrous. N° embryos/pregnant ewe: number of embryos per pregnant ewes.

## 4 Discussion

In the present study, the experimental diets were formulated to ensure an isoenergetic balance between CT and LO groups, as reported by Petit et al. (2004, 2002) in cattle and by Contreras‐Solís et al. (2023) in milked ewes. Consequently, BW and BCS were not affected by the diet, due to the fact that energy intake was homogenous between the CT and LO groups.

Our results indicate that microencapsulated LO allowed PUFAS‐ω3 absorption at the gut level and its subsequent bioavailability. In our study, the increase in PUFA‐ω3 circulating levels was accompanied by a decrease in MUFA, as previously reported in dairy cattle (Zachut et al. 2010). This finding could be attributed to the fact that by‐pass LO protects PUFAs against ruminal biohydrogenation, avoiding/controlling MUFA's increase (Buccioni et al. 2012; Jenkins and Bridges 2007; Jenkins 1993). Also, ruminal biohydrogenation could contribute to reducing the level of the non‐protected MUFA fraction, thus decreasing its plasmatic levels (Buccioni et al. 2012).

The higher plasma concentrations of triglycerides and cholesterol obtained in the LO group in the present study were correlated with higher total EE intake. Triglycerides are mainly provided by fatty acid content in the diet (Bionaz et al. 2020). Nevertheless, cholesterol levels are not directly linked to fat/EE intake because it is synthesized by the animal (Noble 1981; Bionaz et al. 2020). Triglycerides are metabolized at the ruminal level, yielding glycerol and free fatty acids/NEFA to be then re‐esterified in the intestine (Palmquist 1976; Bionaz et al. 2020). At this level, in the LO group, a higher content of PUFAS (from by‐pass LO) could be available to be esterified with glycerol‐3‐phosphate and synthesize triglycerides rich in PUFAS‐ω3 (Pires and Grummer 2008; Bionaz et al. 2020).

The increase of NEFA's level in the LO group found in our study is also reported in previous studies in milking dairy cows (Gonthier et al. 2005) and Merino lambs fed with flaxseed oil (Marino et al. 2018). Gonthier et al. (2005) suggested that this increase could be attributed to other‐ unclear‐ reasons not linked to fatty acids mobilization from adipose tissue, such as excessive fatty acid intake (due to LO supplementation). Thus, it may be suggested that the higher bioavailability of PUFA‐ω3 from the LO group could compete with other fatty acids during triglyceride synthesis at the gut level, producing an excess of NEFA in plasma. This hypothesis needs to be further investigated.

Lipoprotein profile obtained during the peri‐implantation period in this study demonstrates the moderate correlation between total cholesterol and HDL. Cordeiro et al. (2015) also found an increase in cholesterol and HDL during the first days of pregnancy in beef cows supplemented with sunflower seed (rich in PUFAS). In fact, HDL is an effective carrier for cholesterol, which is mobilized toward the liver to be metabolized and reduce its excessive accumulation in body tissues (Oliveira et al. 2021). LDL—as well as HDL—also has an affinity for cholesterol. In addition, LDL could contribute (to a minim degree) together with chylomicrons and very low‐density lipoprotein (VLDL), to distribute triglycerides to peripheral tissues (Bauchart 1993; Mahla et al. 2017; Palmquist and Jenkins 2017).

However, higher levels of triglycerides were not accompanied by higher LDL levels in the LO group. This lower correlation between triglycerides and LDL found in the present study could be attributed to the fact that triglycerides (rich in PUFAS‐ω3) could have more affinity or be more abundant in chylomicrons than VLDL and LDL (Bauchart 1993; Feingold 2022). Thus, chylomicrons could be the main responsible party for PUFAS‐ω3 distribution in our study, as previously suggested (Bauchart 1993; Feingold 2022).

These metabolic changes were, however, not accompanied by differences in follicular function, as evaluated by estradiol levels during the follicular phase. Previous studies (Stocco and Clark 1996; Zachut et al. 2010) suggest that a higher content of PUFAS‐ω6 increases the expression of steroidogenic acute regulatory protein (StAR) in theca cells, which promotes the transfer of cholesterol from the cytosol to the inner mitochondrial membrane and increases the synthesis of estradiol. In our study, LO supplementation did not lead to an increase in PUFAS‐ω6 or estradiol levels. The same finding was also reported in a study performed by Zachut et al. (2010) in dairy cows.

In our study, ovulation rates, as evaluated by the number of CLs per ewe, were not affected by treatment. Hence, the increase in P_4_ levels found in the LO group is likely to be related to a higher steroidogenic capacity of luteal cells. This finding may be explained by the higher cholesterol levels found in the LO group. However, the lack of correlation between cholesterol levels and P_4_ found in our study may indicate that cholesterol may not be totally available for P_4_ synthesis at the luteal level, suggesting an alternative mechanism involved in luteal P_4_ synthesis inside the mitochondrion (Hawkins et al. 1995; Niswender 2002; Hutchinson et al. 2012) or a slow rate of clearance of P_4_ in the supplemented group, such as suggested by Hawkins et al. (1995). This increase was also reported in a preceding study in milked ewes fed with by‐pass LO (Contreras‐Solís et al. 2023), which was attributed to the large size of CL at the onset of the peri‐implantation period.

The higher concentration of P_4_ detected in LO ewes is essential to stimulate the structural and functional changes in uterine tissue to guarantee embryo development (Gray et al. 2001). This also involves the regulation of hormone secretion linked to pregnancy disruption, such as PGF2α synthesis (Arosh et al. 2016).

In this sense, the elevated levels of PUFAS‐ω3 in LO ewes could also be linked to decreased PGF2α synthesis through a lower availability of its precursor—arachidonic acids—as suggested by Mattos et al. (2000). Thus, higher P_4_ levels and lower PGF2α synthesis would contribute to guaranteeing a good embryo–maternal crosstalk during the early stage of pregnancy. Other studies indicate a positive effect of LO on embryo quality in cattle fed with LO; however, most of these studies have been carried out using in vitro embryo production procedures (Zachut et al. 2010; Ponter et al. 2012). In our study, reproductive parameters did not differ between CT and LO ewes. More in vivo and ex vivo studies could be necessary to elucidate the effect of dietary supplementation of LO on embryo quality and its possible effect on endometrial tissue during maternal recognition of pregnancy in ewes.

## 5 Conclusion

The results obtained in this study demonstrated that LO intake increased the plasma levels of PUFAS‐ω3, cholesterol, triglycerides, NEFAS, and HDL. Also, this study demonstrated that luteal function—in terms of P_4_ production—was positively affected by LO treatment. Despite that LO increased the cholesterol levels in plasma, this increase did not impact E_2_ as well as on the percentage of estrus, number of CL, and embryos obtained. Moreover, higher levels of triglycerides and NEFAS observed in the LO group suggest a relationship between higher PUFAS‐ω3 intake and their distribution at systemic and local levels (including reproductive structures). In summary, the results demonstrate that dietary supplementation of LO during the preimplantation period improves luteal function with a possible positive effect on embryo–maternal crosstalk and embryo implantation rate during and after maternal recognition of pregnancy. However, additional studies are needed to elucidate these effects.

## Author Contributions

**Ignacio Contreras‐Solís:** conceptualization (equal), data curation (equal), investigation (equal), methodology (equal), writing – original draft (equal). **Cristian Porcu:** data curation (equal), formal analysis (equal), investigation (equal). **Francesca Daniela Sotgiu:** formal analysis (equal), investigation (equal), writing – original draft (equal). **Valeria Pasciu:** formal analysis (equal). **Neda Todorova:** investigation (equal). **Laura Mara:** investigation (equal). **Fabrizio Chessa:** investigation (equal). **Margherita Addis:** formal analysis (equal). **Myriam Fiori:** formal analysis (equal). **Giovanni Molle:** conceptualization (equal), investigation (equal), methodology (equal), writing – review and editing (equal). **Maria Dattena:** conceptualization (equal), investigation (equal), methodology (equal), writing – review and editing (equal). **Antonio Gonzalez‐Bulnes:** formal analysis (equal). **José Alfonso Abecia:** funding acquisition (equal), writing – review and editing (equal). **Fiammetta Berlinguer:** conceptualization (equal), funding acquisition (equal), investigation (equal), methodology (equal), writing – review and editing (equal).

## Ethics Statement

The procedure of this study is according to Helsinki Declaration guidelines and is approved by the Ethics Committee of AGRIS and UNISS (in accordance with the EU Directive 86/609/EC). The protocol (Protocol‐E8652.2) was approved by the Italian Ministry for Health (Authorization n° 757/2020‐PR).

## Consent

All authors have read and agreed to the published version of the manuscript. All authors have read and approved the final manuscript.

## Conflicts of Interest

The authors declare no conflicts of interest.

## Supporting information


Table S1.

## Data Availability

The data that support the findings of this study are available on request from the corresponding author.
