# Supplementary material for: Microencapsulated Linseed Oil Supplementation Modifies Lipid Profile and Improves Luteal Function in Dairy Sheep
Source: Food Sci Nutr. 2025 Apr 25;13(4):e70097. doi: 10.1002/fsn3.70097 (PMC12031888; doi:10.1002/fsn3.70097)
Supplement: Supplementary file 1 — Table S1. [file FSN3-13-e70097-s001.pdf]

**Table S1.** Spearman rho correlation analyses between Ether extract (EE) intake, plasmatic levels of Low-density (LDL) and High-density lipoproteins and plasmatic levels of Cholesterol, Triglycerides, Non-esterified fatty acid (NEFA) from ewes fed with a control diet without by-pass linseed oil (CT; N = 20) and ewes supplemented with by-pass linseed oil (LO; N = 20).

| | EE | LDL | HDL |
|---|---|---|---|
| Cholesterol | r= 0.612<br>P< 0.01 | r= 0.107<br>P= 0.103 | r= 0.556<br>P< 0.001 |
| Triglycerides | r= 0.648<br>P< 0.001 | r= 0.198<br>P< 0.01 | |
| NEFA | r= 0.338<br>P= 0.106 | | |
